# A cross disciplinary study of link decay and the effectiveness of mitigation techniques

**DOI:** 10.1186/1471-2105-14-S14-S5

**Published:** 2013-10-09

**Authors:** Jason Hennessey, Steven Xijin Ge

**Affiliations:** 1 Department of Mathematics and Statistics, South Dakota State University, Box 2220, Brookings, SD 57007, USA

## Abstract

**Background:**

The dynamic, decentralized world-wide-web has become an essential part of scientific research and communication. Researchers create thousands of web sites every year to share software, data and services. These valuable resources tend to disappear over time. The problem has been documented in many subject areas. Our goal is to conduct a cross-disciplinary investigation of the problem and test the effectiveness of existing remedies.

**Results:**

We accessed 14,489 unique web pages found in the abstracts within Thomson Reuters' Web of Science citation index that were published between 1996 and 2010 and found that the median lifespan of these web pages was 9.3 years with 62% of them being archived. Survival analysis and logistic regression were used to find significant predictors of URL lifespan. The availability of a web page is most dependent on the time it is published and the top-level domain names. Similar statistical analysis revealed biases in current solutions: the Internet Archive favors web pages with fewer layers in the Universal Resource Locator (URL) while WebCite is significantly influenced by the source of publication. We also created a prototype for a process to submit web pages to the archives and increased coverage of our list of scientific webpages in the Internet Archive and WebCite by 22% and 255%, respectively.

**Conclusion:**

Our results show that link decay continues to be a problem across different disciplines and that current solutions for static web pages are helping and can be improved.

## Background

**Figure 1:**
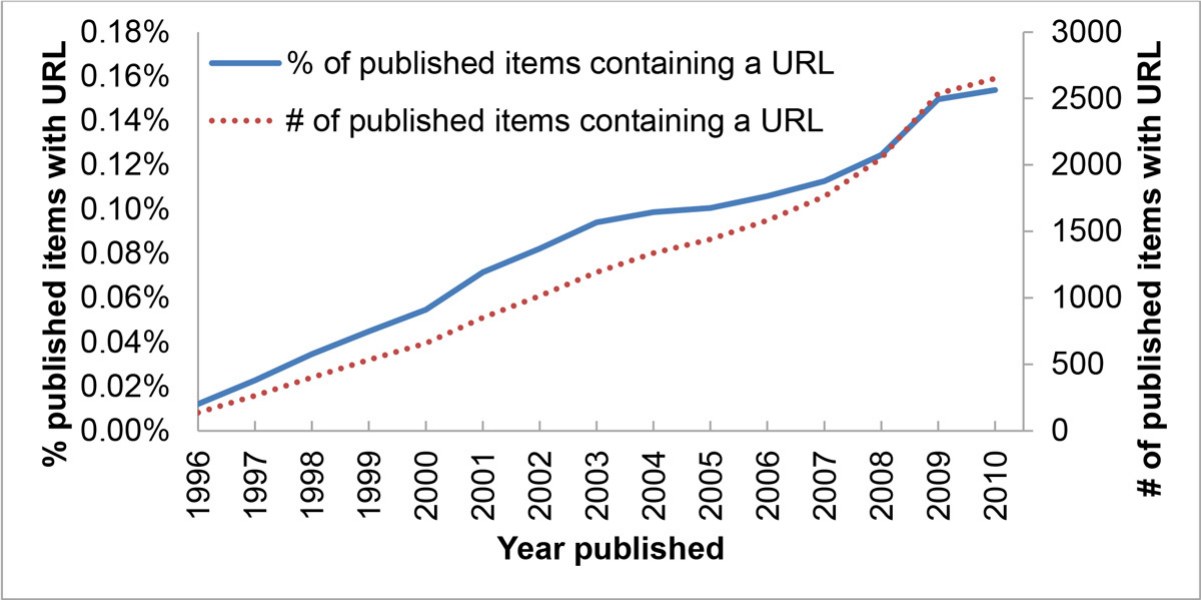
**Growth of scholarly online resources**. Not only are the number of URL-containing articles (those with "http" in the title or abstract) published per year increasing (dotted line), but also the percentage of published items containing URLs (solid line). The annual increase in articles according to a linear fit was 174 with R^2 ^0.97. The linear trend for the percentage was an increase of 0.010% per year with R^2 ^0.98. Source: Thomas Reuter's Web of Science

Scholarly Internet resources play an increasingly important role in modern research. We can see this by the increasing number of URLs published in a paper's title or abstract [1](also see Figure 1). Until now, maintaining the availability of scientific contributions has been decentralized, mature and effective, utilizing methods developed over centuries to archive the books and journals in which they were communicated. As the Internet is still a relatively new medium for communicating scientific thought, the community is still figuring out how best to use it in a way that preserves contributions for years to come. One problem is that continued availability of these online resources is at the mercy of the organizations or individuals that host them. Many disappear after publication (and some even disappear before[2]), leading to a well-documented phenomenon referred to as link rot or link decay.

The problem has been documented in several subject areas, with Table 1 containing a large list of these subject-specific studies. In terms of wide, cross-disciplinary analyses, the closest thus far are those of the biological and medical MEDLINE and PubMed databases by Ducut [1] and Wren [3,4], in addition to Yang's study of the Social Sciences within the Chinese Social Sciences Citation Index (CSSCI) [5].

**Table 1:** Link decay has been studied for several years in specific subject areas.

Field	Links Source/Type	Year(s) of URLs	N	Reference
Biology & Medicine	Science curriculum web links	2000	515	[24]
	
	Full text of 3 dermatology journals	1999-2004	1113	[11]
	
	Sample of bibliographies being published on PubMed	2006	840	[2]
	
	References made in the *Annals of Emergency Medicine*	2000, 2003, 2005	586	[25]
	
	References in 5 biomedical informatics journals.	1999-2004	1049	[26]
	
	MEDLINE titles & abstracts	1994-2006	10208	[1]*
	
	Internet citations in 5 health care management journals from 2002-2004	2009-2010	2011	[14]
	
	MEDLINE abstracts	1995-2007	7462	[3]*

Communications	Citations appearing in research articles in 6 leading communications journals	2000-2003	1600	[27]

Ecology	URLs appearing in the full text of 4 Ecological Society of America journals	1997-2005	2100	[28]

Law	Samples from a collection of born-digital law- and policy-related reports and documents	2007-2010	2372	[29]

Library/Information Science	Citations appearing in 3 leading Information Science journals	1997-2003	2516	[30]
	Sample of citations appearing in library and information science journals	1999-2000	500	[18]

Social Sciences	URLs appearing in the full text of 2 well-respected historical journals	1999-2006	510	[31]
	Citations from articles in the Chinese Social Sciences Index	1998-2007	44973	[5]*

Various	Random Collection of web URLs	1996	371	[15,17]

Various	Citations in 3 highly circulated journals	2002-2003	672	[32]

Various	Supplementary information published in 6 top-cited journals	2000, 2003	585	[33]

Various	Citations from conference articles	1995-2003	1068	[34]

Various Collections				[35-38]

* denotes studies most similar to the current.

Some solutions have been proposed which attack the problem from different angles. The Internet Archive (IA) [6] and WebCite (WC) [7] address the issue by archiving web pages, though their mechanisms for acquiring those pages differ. The IA, beginning from a partnership with the Alexa search engine, employs an algorithm that crawls the Internet at large, storing snapshots of pages it encounters along the way. In contrast, WebCite archives only those pages which are submitted to it, and it is geared toward the scientific community. These two methods, however, can only capture information that is visible from the client. Logic and data housed on the server are not frequently available.

Other tools, like the Digital Object Identifier (DOI) System [8] and Persistent Uniform Resource Locator (PURL) [9], provide solutions for when a web resource is moved to a different URL but is still available. The DOI System was created by an international consortium of organizations wishing to assign unique identifiers to items such as movies, television shows, books, journal articles, web sites and data sets. It encompasses several thousand "Naming Authorities" organized under a few "Registration Agencies" that have a lot of flexibility in their business models[10]. Perhaps 30-60% of link rot could be solved using DOIs and PURLs[11,12]. However they are not without pitfalls. One is that a researcher or company could stop caring about a particular tool for various reasons and thus not be interested in updating its permanent identifier. Another is that the one wanting the permanent URL (the publishing author) is frequently not the same as the person administering the site itself over the long term, thus we have an imbalance of desire vs. responsibilities between the two parties. A third in the case of the DOI System is that there may be a cost in terms of money and time associated with registering their organization that could be prohibitive to authors that don't already have access to a Naming Authority[1]. One example of a DOI System business model would be that of the California Digital Library's EZID service, which charges a flat rate (currently $2,500 for a research institution) for up to 1 million DOIs per year[13].

In this study, we ask two questions: what are the problem's characteristics in scientific literature as a whole and how is it being addressed? To assess progress in combating the problem, we evaluate the effectiveness of the two most prevalent preservation engines: and examine the effectiveness of one prototyped solution. If a URL is published in the abstract, it is assumed that the URL plays a prominent role within that paper, similar to the rationale proposed by Wren [4].

## Results

**Figure 2:**
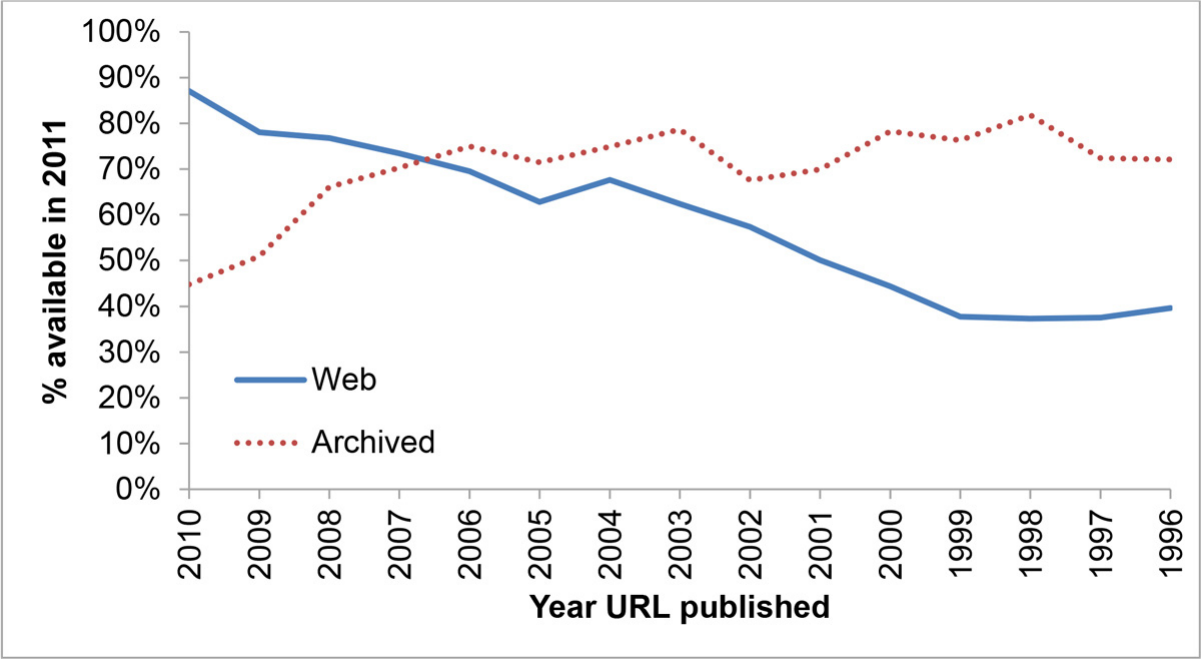
**The accessibility of URLs from a particular year is closely correlated with age**. The probability of being available (solid line) declines by 3.7% every year based on a linear model with R^2 ^0.96. The surveyed archival engines have about a 70-80% archival rate (dotted line) following an initial ramp time.

**Figure 3:**
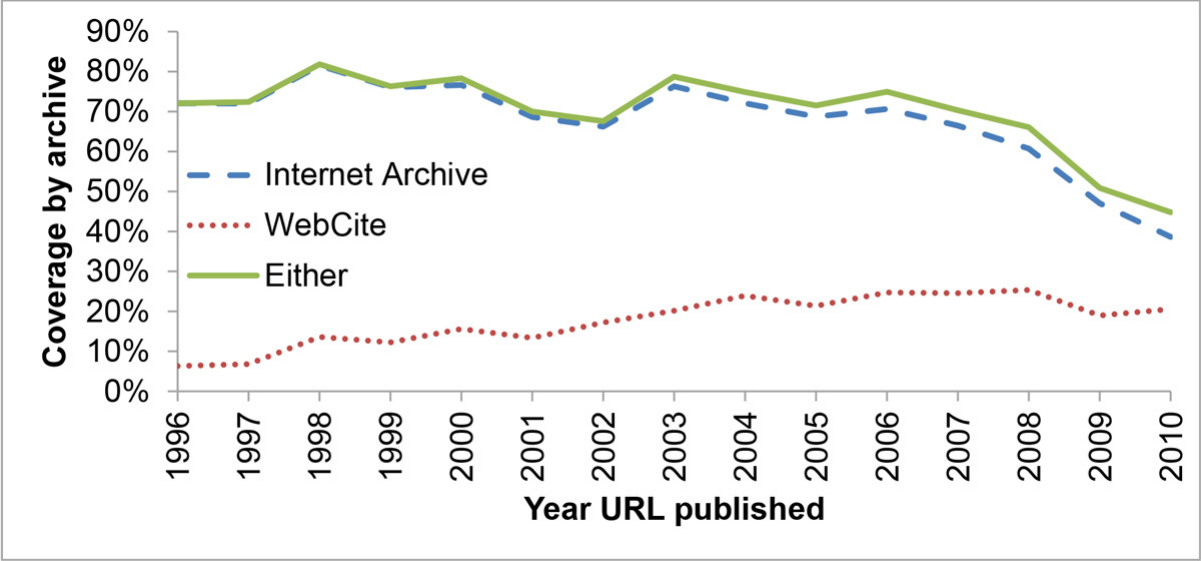
**URL presence in the archives**. Percentage of URLs found in the archives of the Internet Archive (dashed line), WebCite (dotted line) or in any group (solid line). IA is older, and thus accounts for the lion's share of earlier published URLs, though as time goes on WebCite is offering more and more.

Our goals are to provide some metrics that are useful in understanding the problem of link decay in a cross-disciplinary fashion and to examine the effectiveness of the existing archival methods while proposing some incremental improvements. To accomplish these tasks, we downloaded 18,231 Web of Science (WOS) abstracts containing "http" in the title or abstract from the years under study (1996-2010), out of which 17,110 URLs (14,489 unique) were extracted and used. We developed Python scripts to access these URLs over a 30-day period. For the period studied, 69% of the published URLs (67% of the unique) were available on the live Internet, the Internet Archive's Wayback Machine had archived 62% (59% unique) of the total and WebCite had 21% (16% unique). Overall, 65% of all URLs (62% unique) were available from one of the two surveyed archival engines. Figure 2 contains a breakdown by year for availability on the live web as well as through the combined archives, and Figure 3 illustrates each archival engine's coverage. The median lifetime for published URLs was found to be 9.3 years (95% CI [9.3,10.0]), with the median lifetime amongst unique URLs also being 9.3 years (95% CI [9.3,9.3]). Subject-specific lifetimes may be found in Table 2. Using a simple linear model, the chances that a URL published in a particular year is still available goes down by 3.7% for each year added to its age with an R^2 ^of 0.96. Its chances of being archived go up after an initial period of flux (see Figure 2). Submitting our list of unarchived but living URLs to the archival engines showed dramatic promise, increasing the Internet Archive's coverage of the dataset by 2080 URLs, an increase of 22%, and WebCite's by 6348, an increase of 255%.

**Table 2:** Comparison of certain statistics based on the subject of a given URL.

Subject	Total	# Alive (%)	Median Survival with 95% CI in years
Biochemistry & Molecular Biology	4585	3231 (70%)	10.8 (9.0,11.0)

Biotechnology & Applied Microbiology	2225	1586 (71%)	9.0 (8.8,9.0)

Computer Science	2073	1225 (59%)	8.3 (7.0,9.0)

Biochemical Research Methods	2023	1463 (72%)	8.5 (8.5,8.6)

Mathematical & Computational Biology	1661	1200 (72%)	7.5 (7.5,9.0)

Genetics & Heredity	1302	914 (70%)	8.8 (8.8,10.0)

Physics	809	458 (57%)	8.0 (7.6,9.0)

Engineering	703	419 (60%)	7.2 (7.1,10.5)

Statistics & Probability	699	440 (63%)	7.6 (7.0,9.0)

Chemistry	591	397 (67%)	11.4 (9.0,11.9)

Biophysics	432	270 (63%)	10.1 (10.1,10.1)

Astronomy & Astrophysics	416	268 (64%)	11.3 (11.1,NA)

Mathematics	406	254 (63%)	10.7 (4.5,NA)

Zoology	357	319 (89%)	11.2 (9.6,NA)

Cell Biology	353	242 (69%)	8.0 (8.0,10.8)

Biology	346	242 (70%)	9.8 (7.3,NA)

Oncology	342	239 (70%)	6.9 (6.9,7.0)

Plant Sciences	315	235 (75%)	9.8 (8.2,NA)

Environmental Sciences	304	190 (63%)	8.0 (7.6,9.5)

Medicine	293	219 (75%)	13.3 (10.0,NA)

Subjects are assigned to journals and not specific papers. Note that in these models, a given URL could contribute to multiple subjects due to appearing in multiple journals which could also have multiple subject areas. Where possible, specific subjects were generalized (for example, "Computer Science, Interdisciplinary Applications" became "Computer Science"). Median survival estimated using R's survfit(). "NA" indicates that an upper 95% limit was unable to be computed.

How common are published, scholarly online resources? For WOS, both the percentage of published items which contained a URL as well as their absolute number increased steadily since 1996 as seen in Figure 1. Simple linear fits showed the former's annual increase at a conservative 0.010 % per year with an R^2 ^of 0.98 while the latter's increase was 174 papers with an R^2 ^of 0.97.

A total of 189 (167 unique) DOI URLs were identified, consisting of 1% of the total, while 9 PURLs (8 unique) were identified. Due to cost[14], it is likely that DOIs will remain useful for tracking commercially published content though not the scholarly online items independent of those publishers.

### URL survival

In order to shed some light on the underlying phenomena of link rot, a survival regression model was fitted with data from the unique URLs. This model, shown in Table 3, identified 17 top-level domains, the number of times a URL has been published, a URL's directory structure depth (hereafter referred to as "depth", using the same definition as [15]), the number of times the publishing article(s) has been cited, whether articles contain funding text as well as 4 journals as having a significant impact on a URL's lifetime at the P< 0.001 level. This survival regression used the logistic distribution and is interpreted similarly to logistic models. To determine the predicted outcome for a particular URL, one takes the intercept (5.2) and adds to it the coefficients for the individual predictors if those predictors are different from the base level; coefficients here are given in years. If numeric, one first multiplies before adding. The result is then interpreted as the location of the peak of a bell curve for the expected lifetime, instead of a log odds ratio as a regular logistic model would give. Among the two categorical predictors (domains and journals having more than 100 samples), the three having the largest positive impact on lifetimes were the journal *Zoological Studies *(+16) and the top-level domains *org *and *dk *(+8 for both). Though smaller in magnitude than the positive ones, the 3 categorical predictors having the largest negative impact were the journals *Computer Physics Communications *(-4) and *Bioinformatics *(-2) as well as the domain *kr *(-3), though the P values associated with the latter two are more marginal than some of the others (.006 and .02 respectively).

**Table 3:** Results of fitting a parametric survival regression using the logistic distribution to the unique URLs.

Variable	Value	p	5%	95%
(Intercept)	5.22	3.3E-30	4.46	5.97

Log2(URL published)	3.57	1.4E-17	2.88	4.25

depth	-1.46	7.0E-32	-1.66	-1.25

Log2(TimesCited + 1)	0.25	2.8E-04	0.13	0.36

Funding text present	3.43	2.8E-11	2.59	4.28

**Domain**				

au	4.53	1.5E-04	2.56	6.49

be	3.31	1.9E-02	0.99	5.64

ca	4.88	1.7E-06	3.20	6.56

ch	6.45	7.2E-08	4.48	8.42

cn	1.50	1.3E-01	-0.13	3.13

com	6.02	2.2E-18	4.89	7.16

de	5.74	6.1E-16	4.57	6.91

dk	7.66	5.7E-07	5.14	10.18

edu	3.77	1.6E-13	2.93	4.61

es	3.05	5.4E-03	1.25	4.85

fr	3.65	6.6E-07	2.44	4.85

gov	5.51	1.2E-15	4.38	6.64

il	5.92	3.6E-04	3.19	8.65

in	4.78	2.2E-04	2.65	6.91

it	5.51	1.4E-08	3.91	7.11

jp	5.07	8.0E-09	3.62	6.51

kr	-3.35	2.0E-02	-5.73	-0.97

net	7.01	4.2E-11	5.26	8.76

nl	6.78	1.1E-06	4.49	9.07

org	8.10	2.4E-36	7.04	9.16

ru	3.90	2.3E-03	1.80	6.01

se	1.71	2.4E-01	-0.69	4.12

tw	1.64	1.7E-01	-0.33	3.61

uk	4.49	4.2E-12	3.42	5.56

**Source**				

Bioinformatics	-2.04	5.7E-03	-3.25	-0.83

BMC Bioinformatics	2.69	3.9E-05	1.62	3.77

BMC Genomics	0.88	4.7E-01	-1.13	2.89

Comp. Physics Comm.	-4.00	3.0E-05	-5.57	-2.42

Genome Research	0.56	7.1E-01	-1.92	3.04

Nucleic Acids Research	1.28	8.6E-04	0.65	1.91

PLoS ONE	-0.39	8.0E-01	-2.95	2.18

Zoological Studies	16.42	2.2E-15	13.01	19.83

Positive numbers indicate longer median lifetimes. Much like a logistic model, coefficients can be added to the intercept value (after multiplying in the case of numeric predictors) to obtain a median lifetime. For example, the median expected lifetime for a URL published once, with depth 0, whose publishing article had 1 citation, no funding text, domain au and published in a Journal not listed (ie- in the default) would be: (Intercept) 5.22 + Log2(1)*3.57 + 0*-1.46 + Log2(1+1)*0.25 + 0*3.43 + 4.53 = 10 years

### Predictors of availability

**Figure 4:**
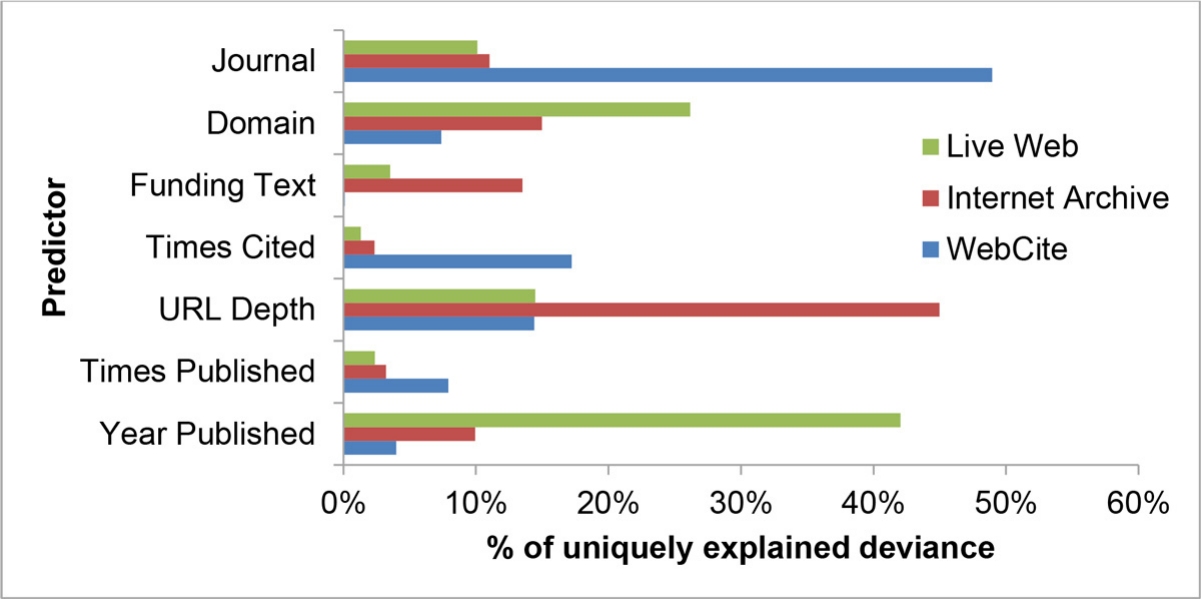
**How important is each predictor in predicting whether a URL is available?** This graph compares what portion of the overall deviance is explained uniquely by each predictor for each of the measured outcomes. A similar list of predictors (differing only in whether the first or last year a URL was published) without interaction terms was employed to construct 3 logistic regression models. The dependent variable for each of the outcomes under study (Live Web, Internet Archive and WebCite) was availability at the time of measurement. Unique deviance was calculated by dropping each term and measuring the change in explained deviance in the logistic model. Results were then expressed as a percentage of the total uniquely explained deviance for each of the 3 methods.

While examining URL survival and archival, it is not only interesting to ask which factors significantly correlate with a URL lasting but also which account for most of the differences. To that end, we fit logistic models for each of the measured outcomes (live web, Internet Archive and Web Citation availabilities) to help tease out that information. To enhance comparability, a similar list of predictors (differing only in whether the first or last year a URL was published was used) without interaction terms was employed for all 3 methods and unique deviance calculated by dropping each term from the model and measuring the change in residual deviance. Results were then expressed as a percentage of the total uniquely explained deviance and are graphically shown in Figure 4.

For live web availability, the most deviance was explained by the last year a URL was published (42%) followed by the domain (26%). That these two predictors are very important agrees with much of the published literature thus far. For the Internet Archive, by far the most important predictor was the URL depth at 45%. Based on this, it stands to reason that the Internet Archive either prefers more popular URLs which happen to be at lower depths or employs an algorithm that prioritizes breadth over depth. Similar to the IA, WC had a single predictor that accounted for much of the explained deviance, with the publishing journal representing 49% of the explained deviance. This may reflect WC's efforts to work with publishers as the model shows one of the announced early adopters, BioMed Central [7], as having the two measured journals (BMC Bioinformatics and BMC Genomics) with the highest retention rates. Therefore, WC is biased towards a publication's source (journals).

### Archive site performance

Another way to measure the effectiveness of the current solutions to link decay is to look at the number of "saved" URLs, or those missing ones that are available through archival engines. Out of the 31% of URLs (33% of the unique) which were not accessible on the live web, 49% of them (47% of the unique) were available in one of the two engines, with IA having 47% (46% unique) and WC having 7% (6% unique). WC's comparatively lower performance can likely be attributed to a combination of its requirement for human interaction and its still-growing adoption.

In order to address the discrepancy, all sites that were still active but not archived were submitted to the engine(s) from which they were missing. Using the information gleaned from probing the sites as well as the archives, URLs missing from one or both of the archives, yet still alive, were submitted programmatically. This included submitting 2,662 to the Wayback Machine as well as 7,477 to WebCite, of which 2,080 and 6,348 were successful, respectively.

## Discussion

### Submission of missing URLs to archives

Archiving missing URLs in each of the archival engines had their own special nuances. For the Internet Archive, the lack of a practical documented way of submitting URLs (see http://faq.web.archive.org/my-sites-not-archived-how-can-i-add-it/) necessitated trusting a message shown by the Wayback Machine when one finds a URL that isn't archived and clicks the "Latest" button. In this instance, the user is sent to the URL "http://liveweb.archive.org/<url>" which has a banner proclaiming that the page "will become part of the permanent archive in the next few months". Interestingly, as witnessed by requests for a web page hosted on a server for which the authors could monitor the logs, only those items requested by the client were downloaded. This meant that if only a page's text were fetched, supporting items such as images and CSS files would not be archived. To archive the supporting items and avoid duplicating work, wget's "--page-requisites" option was used instead of a custom parser.

WebCite has an easy-to-use API for submitting URLs, though limitations during the submission of our dataset presented some issues. The biggest issue was WebCite's abuse detection process, which would flag the robot after it had made a certain number of requests. To account for this and be generally nice users, we added logic to ensure a minimum delay between archival requests submitted to both the IA and WC. Exponential delay logic was implemented for WC when encountering general timeouts, other failures (like mysql error messages) or the abuse logic. Eventually, we learned that certain URLs would cause WC's crawler to timeout indefinitely, requiring the implementation of a maximum retry count (and a failure status) if the error wasn't caused by the abuse logic.

To estimate what impact we had on the archives' coverage of the study URLs, we compared a URL survey done directly prior to our submission process to one done afterwards; a period of about 3.5 months. It was assumed that the contribution due to unrelated processes would not be very large given that there was only a modest increase in coverage, 5% for IA and 1% for WC, over the previous period of just under a year and a half.

Each of the two archival engines had interesting behaviors which required gauging successful submission of a URL by whether it was archived as of a subsequent survey rather than using the statuses returned by the engines. For the Internet Archive, it was discovered that an error didn't always indicate failure, as there were 872 URLs for which wget returned an error but which were successfully archived. Conversely, WebCite returned an asynchronous status, such that even in the case of a successful return the URL might fail archival; the case in 955 out of a total of 7,285.

Submitting the 2662 URLs to IA took a little less than a day, whereas submitting 7285 to WC took over 2 months. This likely reflects IA's large server capacity, funding and platform maturity due to its age.

### Generating the list of unique URLs

Converting some of the potential predictors from the list of published URLs to the list of unique URLs presented some unique issues. In particular, while converting those based on the URL itself (domain, depth, whether alive or in an archive) were straightforward, those which depended upon a publishing article (number of times URL was published, the number of times an article was cited, publishing journal, whether there was funding text) were estimated by collating the data from each publishing. Only a small amount, 8%, of the unique URLs, appeared more than once, and among the measured variables that pertained to the publishing there was not a large amount of variety. Amongst repeatedly-published URLs, 43% appeared in only one journal and the presence of funding text was the same 76% of the time. For calculating the number of times a paper was published, multiple appearances of a URL within a given title/abstract were counted as one. Thus, while efforts were made to provide a representative collated value where appropriate, it's expected that different methods would not have produced significantly different results.

### Additional sources of error

Even though WOS's index appears to have better quality Optical Character Recognition (OCR) than PubMed, it still has OCR artifacts. To compensate for this, the URL extraction script tried to use some heuristics to detect the most common sources of error and correct them. Some of the biggest sources of error were: randomly inserted spaces in URLs, "similar to" being substituted for the tilde character, periods being replaced with commas and extra punctuation being appended to the URL (sometimes due to the logic added to address the first issue).

Likely the largest contributors to false negatives are errors in OCR and the attempts to compensate for them. In assessing the effectiveness of our submissions to IA, it is possible that the estimate could be understated due to URLs that had been submitted but not yet made available within the Wayback Machine.

Dynamic websites with interactive content, if only present via an archiving engine, would be a source of false positives, as the person accessing the resource would presumably want to use it as opposed to viewing the design work of its landing page. If a published web site goes away and another installed in its place (especially true if a .com or .net domain is allowed to expire), then the program will not be able to tell the difference since it will see a valid (though impertinent) web site. In addition, though page contents can change and lose relevance from their original use[16], dates of archival were not compared to the publication date.

Another source of false positive error would be uncaught OCR artifacts that insert spaces within URLs if it truncated the path but left the correct host intact. The result would be a higher probability that the URL would appear as a higher level index page, which are generally more likely to function than pages at lower levels [11,12].

### Bibliographic database

Web of Science was chosen because, compared to PubMed, it was more cross-sectional and had better OCR quality based on a small sampling. Many of the other evaluation criteria were similar between PubMed and WOS, as both contain scholarly work and have an interface to download bibliographic data. Interestingly, due to the continued presence of OCR issues in newer articles, it appears that bibliographic information for some journals is not yet passed electronically.

## Conclusions

Based on the data gathered in this and other studies, it is apparent that there is still a problem with irretrievable scholarly research on the Internet. We found that roughly 50% of URLs published 11 years prior to the survey (in 2000) are still left standing. Interesting is that the rate of decay for late-published URLs (within the past 11 years) appears to be higher than that for the older ones, lending credence to what Koehler suggested about eventual decay rate stabilization[17]. Survival rates for living URLs published between 1996 and 1999, inclusive, only vary by 2.4% (1.5% for unique) and have poor linear fits (R^2 ^of .51 and .18 for unique), whereas years [2000, 2010] have linear slope 0.031 and R^2 ^.90 (.036 and R^2 ^.95 for unique URLs using the first published year) indicating that the availability between years for older URLs is much more stable whereas the availability for more recent online resources follow a linear trend with a predictable loss rate. Overall, 84% of URLs (82% of the unique) were available in some manner: either via the web, IA or WC.

**Figure 5:**
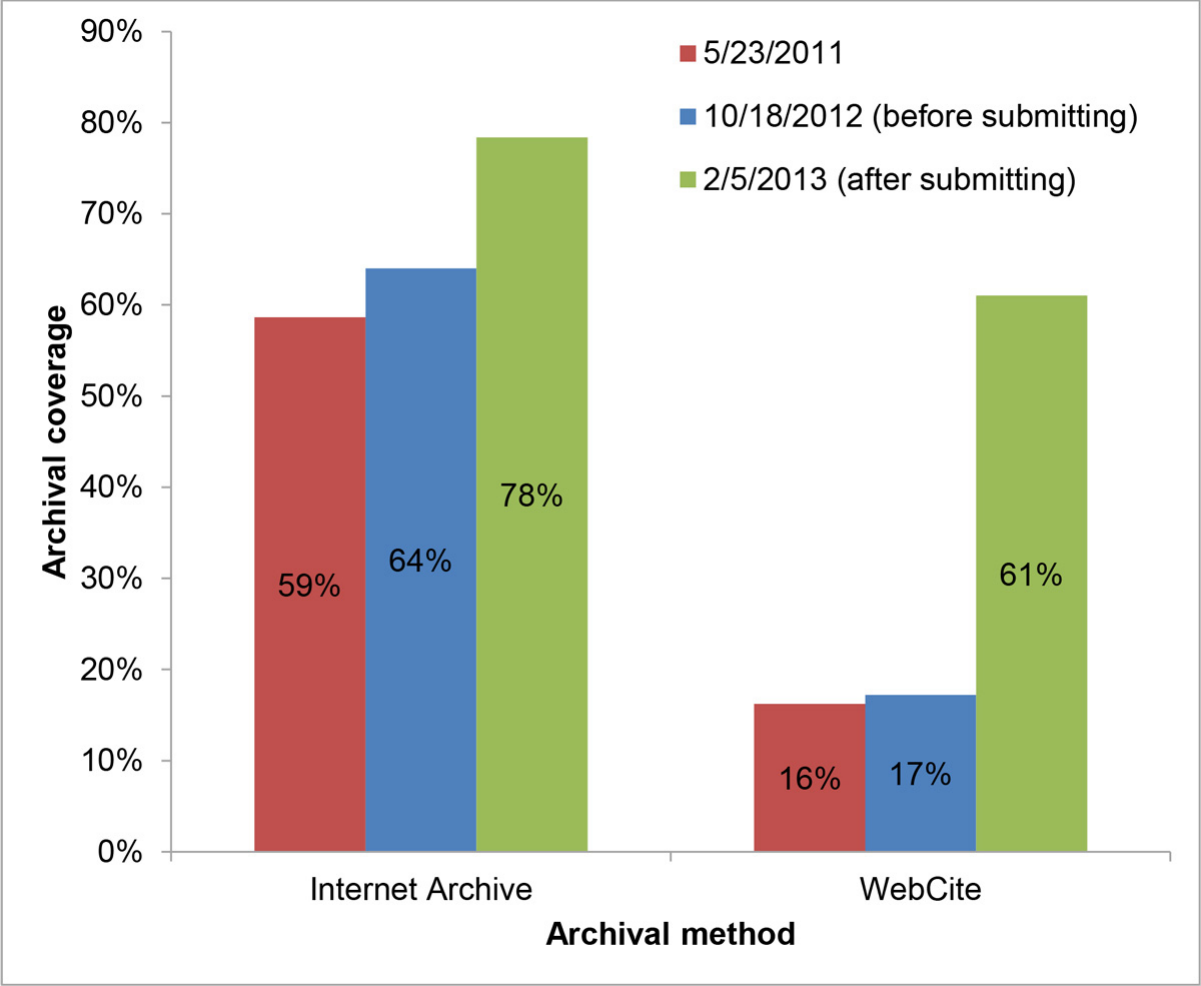
**Coverage of the scholarly URL list for each archival engine at different times**. All URLs marked as alive in 2011 but missing from an archive were submitted between the 2012 and 2013 surveys. The effect of submitting the URLs is most evident in the WebCite case though the Internet Archive also showed substantial improvement. Implementing an automated process to do this could vastly improve the retention of scholarly static web pages.

Several remedies are available to address different aspects of the link decay problem. For data-based sites that can be archived properly with an engine such as the Internet Archive or WebCite, one remedy is to submit the missing sites which are still alive to the archiving engines. Based on the results of our prototype (illustrated in Figure 5), this method was wildly successful, increasing IA's coverage of the study's URLs by 22% and WebCite's by 255%. Journals could require authors to submit URLs to both the Internet Archive and WebCite, or alternatively programs similar to those employed in this study could be used to do it automatically. Another way to increase archival would be for the owners of published sites to ease restrictions for archiving engines since 507 (352 unique) of the published URLs had archiving disabled via robots.txt according to the Internet Archive. Amongst these, 16% (22% of the unique) have already ceased being valid. While some sites may have good reason for blocking automated archivers (such as dynamic content or licensing issues), there may be others that could remove their restrictions entirely or provide an exception for preservation engines.

To address the control issue for redirection solutions (DOI, PURL) mentioned in the introduction, those who administer cited tools could begin to maintain and publish a permanent URL on the web site itself. Perhaps an even more radical step would be for either these existing tools or some new tool to take a Wikipedia approach and allow end-users to update and search a database of permanent URLs. Considering the studies that have shown around at least 30% of dead URLs to be locatable using web search engines [3,18], such a peer-maintained system could be effective and efficient, though spam could be an issue if not properly addressed.

For dynamic websites, the current solutions are more technically involved, potentially expensive and less feasible. These include mirroring (hosting a website on another server, possibly at another institution) and providing access to the source code, both of which require time and effort. Once the source is acquired, it can sometimes take considerable expertise to make use of it as there may be complex libraries or framework configuration, local assumptions hard-coded into the software or it could be written for a different platform (GPU, Unix, Windows, etc.). The efforts to have reproducible research, where the underlying logic and data behind the results of a publication are made available to the greater community, have stated many of the same requirements as preserving dynamic websites [19,20]. Innovation in this area could thus have multiple benefits beyond just the archival.

## Methods

### Data preparation and analysis

The then-current year (2011) was excluded to eliminate bias from certain journals being indexed sooner than others. For analysis and statistical modeling, the R program [21] and its "survival" library [22] were used (scripts included in Additional file 1).

Wherever possible, statistics are presented in 2 forms: one representing the raw list of URLs extracted from abstracts and the other representing a deduplicated set of those URLs. The former is most appropriate when thinking about what a researcher would encounter when trying to use a published URL in an article of interest and also serves as a way to give weight to multiply-published URLs. The latter is more appropriate when contemplating scholarly URLs as a whole or when using statistical models that assume independence between samples.

URLs not the goal of this study such as journal promotions and invalid URLs were excluded using computational methods as much as possible in order to minimize subjective bias. The first method, removing 943 (26 unique), looked for identical URLs which comprised a large percentage of a journal's published collection within a given year. Upon manual examination, a decision was then made whether to eliminate them. The second method, which identified 18 invalid URLs (all unique), consisted of checking for WebCitation's "UnexpectedXML" error. These URLs were corrupted to the point that they interfered with XML interpretation of the request due either to an error in our parsing or the OCR.

DOI sites were identified by virtue of containing "http://dx.doi.org". PURL sites were identified by virtue of containing "http://purl." in the URL. Interestingly, 3 PURL servers were identified through this mechanism: http://purl.oclc.org, http://purl.org and http://purl.access.gpo.gov.

To make for results more comparable to prior work as well as easier to interpret analysis, a URL was considered available if it successfully responded to at least 90% of the requests and unavailable if less than that. This method is similar to the method used by Wren[4], and differs from Ducut's[1] by not using a "variable availability" category defined as being available > 0% and < 90% of the time. Our results show that 466 unique URLs (3.2%) would have been in this middle category, a number quite similar to what Wren's and Ducut's would have been (3.4% and 3.2%, respectively). Being such a small percentage of the total, their treatment is not likely to affect analysis much regardless of how they are interpreted. Having binary data also eases interpretation of the statistical models. In addition, due to the low URL counts for 1994 (3) and 1995 (22), these years were excluded from analysis.

#### Survival model

Survival analysis was chosen to analyze living URLs due to its natural fit; like people, URLs have lifetimes and we are interested in discussing them, what causes them to be longer or shorter and by how much. Lifetimes were calculated by assuming URLs were alive each time they were published, which is a potential source of error [2]. Data was coded as either right or left-censored; right-censored since living URLs presumably would die at an unknown time in the future and left-censored because it was unknown when a non-responding URL had died. Ages were coded in months rather than years in order to increase accuracy and precision.

Parametric survival regression models were constructed using R's *survreg()*. In selecting the distribution to use, all of those available were tried, with the logistical showing the best overall fit based on Akaike Information Criterion (AIC) score. Better fits for two of the numeric predictors (number of citations to a publishing paper and number of times a URL was published) were obtained by taking the base 2 logarithm. Collinearity was checked by calculating the variance inflation factor against a logistic regression fit to the web outcome variable. Overall lifetime estimates were made using the *survfit() *function from R's survival library.

#### Extracting and testing URLs

To prepare a list of URLs (and their associated data), a collection of bibliographic data was compiled by searching WOS for "http" in the title or abstract, downloading the results (500 at a time), then finally collating them into a single file. A custom program (extract_urls.py in Additional file 1) was then used to extract the URLs and associated metadata from these, after which 5 positive and 2 negative controls were added. A particular URL was only included once per paper.

With the extracted URLs in hand, another custom program (check_urls_web.py in Additional file 1) was used to test the availability of the URLs 3 times a day over the course of 30 days, starting April 16, 2011. These times were generated randomly by scheduler.py (included in Additional file 1), the algorithm guaranteeing that no consecutive runs were closer than 2 hours. A given URL was only visited once per run even if it was published multiple times, saving load on the server and speeding up the total runtime (which averaged about 25 minutes due to use of parallelism). Failure was viewed as anything that caused an exception in python's "urllib2" package (which includes error statuses, like 404), with the exception reason being recorded for later analysis.

While investigating some of the failed fetches, a curious thing was noted: there were URLs that would consistently work with a web browser but not with the Python program or other command line downloaders like wget. After some investigation, it was realized that the web server was denying access to unrecognized User Agent strings. In response, the Python program adopted the User Agent of a regular browser and subsequently reduced the number of failed URLs.

At the end of the live web testing period, a custom program (check_urls_archived.py in Additional file 1) was used to programmatically query the archive engines on May 23, 2011. For the Internet Archive's Wayback Machine, this was done using an HTTP HEAD request (which saves resources vs. GET) on the URL formed by "http://web.archive.org/web/*/" + <the url>. Status was judged by the resulting HTTP status code with 200 meaning success, 404 meaning not archived, 403 signifying a page blocked due to robots.txt and 503 meaning that the server was too busy. Because there were a number of these 503 codes, the script would make up to 4 attempts to access the URL, with increasing back off delays to keep from overloading IA's servers. The end result still contained 18, which were counted as not archived for analysis. For WebCite, the documented API was used. This supports returning XML, a format very suitable to automated parsing [23]. For sites containing multiple statuses, any successful archiving was taken as a success.

## Competing interests

The authors declare that they have no competing interests.

## Authors' contributions

JH implemented the tools for data acquisition and statistical analysis as well as performed a literature review and drafting of the paper. SXG implemented an initial prototype and provided valuable feedback at every step of the process, including critical revision of this manuscript.

## Supplementary Material

Additional file 1 **supplement.zip**. Contains source code used to perform the study, written in python and R. README.txt contains descriptions for each file. Click here for file

## Declarations

Publication of this article was funded by the National Institutes of Health [GM083226 to SXG].

This article has been published as part of *BMC Bioinformatics *Volume 14 Supplement 14, 2013: Proceedings of the Tenth Annual MCBIOS Conference. Discovery in a sea of data. The full contents of the supplement are available online at http://www.biomedcentral.com/bmcbioinformatics/supplements/14/S14.
